# Is diet partly responsible for differences in COVID-19 death rates between and within countries?

**DOI:** 10.1186/s13601-020-00323-0

**Published:** 2020-05-27

**Authors:** Jean Bousquet, Josep M. Anto, Guido Iaccarino, Wienczyslawa Czarlewski, Tari Haahtela, Aram Anto, Cezmi A. Akdis, Hubert Blain, G. Walter Canonica, Victoria Cardona, Alvaro A. Cruz, Maddalena Illario, Juan Carlos Ivancevich, Marek Jutel, Ludger Klimek, Piotr Kuna, Daniel Laune, Désirée Larenas-Linnemann, Joaquim Mullol, Nikos G. Papadopoulos, Oliver Pfaar, Boleslaw Samolinski, Arunas Valiulis, Arzu Yorgancioglu, Torsten Zuberbier, The ARIA group, Amir Hamzah Abdul Latiff, Baharudin Abdullah, Werner Aberer, Nancy Abusada, Ian Adcock, Alejandro Afani, Ioana Agache, Xenofon Aggelidis, Jenifer Agustin, Cezmi Akdis, Mübeccel Akdis, Mona Al-Ahmad, Abou Al-Zahab Bassam, Oscar Aldrey-Palacios, Emilio Alvarez Cuesta, Ashraf Alzaabi, Salma Amad, Gene Ambrocio, Isabella Annesi-Maesano, Ignacio Ansotegui, Josep Anto, Hasan Arshad, Maria Cristina Artesani, Estrella Asayag, Francesca Avolio, Khuzama Azhari, Ilaria Baiardini, Nissera Bajrović, Petros Bakakos, Sergio Bakeyala Mongono, Christine Balotro-Torres, Sergio Barba, Cristina Barbara, Elsa Barbosa, Bruno Barreto, Joan Bartra, Eric D. Bateman, Lkhagvaa Battur, Anna Bedbrook, Martín Bedolla Barajas, Bianca Beghé, Elizabeth Bel, Ali Ben Kheder, Mikael Benson, Camelia Berghea, Karl-Christian Bergmann, David Bernstein, Mike Bewick, Slawomir Bialek, Artur Białoszewski, Thomas Bieber, Nils Billo, Maria Beatrice Bilo, Carsten Bindslev-Jensen, Leif Bjermer, Hubert Blain, Malgorzata Bochenska Marciniak, Christine Bond, Attilio Boner, Matteo Bonini, Sergio Bonini, Sinthia Bosnic-Anticevich, Isabelle Bosse, Sofia Botskariova, Jacques Bouchard, Louis-Philippe Boulet, Rodolphe Bourret, Philippe Bousquet, Fulvio Braido, Andrew Briggs, Christopher Brightling, Jan Brozek, Roland Buhl, Roxana Bumbacea, María Teresa Burguete Cabañas, Andrew Bush, William W. Busse, Jeroen Buters, Fernan Caballero-Fonseca, Moïses A. Calderon, Mario Calvo, Paulo Camargos, Thierry Camuzat, Antonio Cano, G. Walter Canonica, Arnaldo Capriles-Hulett, Luis Caraballo, Vicky Cardona, Kai-Hakon Carlsen, Jorge Caro, Warner Carr, Fredelita Carreon-Asuncion, Ana Maria Carriazo, Thomas Casale, Mary Ann Castor, Elizabeth Castro, Lorenzo Cecchi, Alfonso Cepeda Sarabia, Ramanathan Chandrasekharan, Yoon-Suk Chang, Victoria Chato-Andeza, Lida Chatzi, Christina Chatzidaki, Niels H. Chavannes, Yuzhi Chen, Lei Cheng, Tomas Chivato, Ekaterine Chkhartishvili, George Christoff, Henry Chrystyn, Derek K. Chu, Antonio Chua, Alexander Chuchalin, Kian Fan Chung, Alberto Cicerán, Cemal Cingi, Giorgio Ciprandi, Ieva Cirule, Ana Carla Coelho, Jannis Constantinidis, Jaime Correia de Sousa, Elisio Costa, David Costa, María del Carmen Costa Domínguez, André Coste, Linda Cox, Alvaro A. Cruz, John Cullen, Adnan Custovic, Biljana Cvetkovski, Wienczyslawa Czarlewski, Gennaro D’Amato, Jane da Silva, Ronald Dahl, Sven-Erik Dahlen, Vasilis Daniilidis, Louei Darjazini Nahhas, Ulf Darsow, Frédéric de Blay, Eloisa De Guia, Chato de los Santos, Esteban De Manuel Keenoy, Govert De Vries, Diana Deleanu, Pascal Demoly, Judah Denburg, Philippe Devillier, Alain Didier, Maria Dimou, Anh Tuan Dinh-Xuan, Ratko Djukanovic, Dejan Dokic, Margarita Gabriela Domínguez Silva, Habib Douagui, Nikolaos Douladiris, Maria Doulaptsi, Gérard Dray, Ruta Dubakiene, Stephen Durham, Mark Dykewicz, Didier Ebo, Natalija Edelbaher, Patrik Eklund, Yehia El-Gamal, Zeinab A. El-Sayed, Shereen S. El-Sayed, Magda El-Seify, Regina Emuzyte, Lourdes Enecilla, Heidilita Espinoza, Jesús Guillermo Espinoza Contreras, John Farrell, Lenora Fernandez, Antje Fink Wagner, Alessandro Fiocchi, Wytske J. Fokkens, Jean-François Fontaine, Francesco Forastiere, Jose Miguel Fuentes Pèrez, Emily Gaerlan–Resureccion, Mina Gaga, José Luis Gálvez Romero, Amiran Gamkrelidze, Alexis Garcia, Cecilia Yvonne García Cobas, María de la Luz Hortensia García Cruz, Jacques Gayraud, Bilun Gemicioglu, Sonya Genova, José Gereda, Roy Gerth van Wijk, Maximiliano Gomez, Sandra González Diaz, Maia Gotua, Christos Grigoreas, Ineta Grisle, Marta Guidacci, Nick Guldemond, Zdenek Gutter, Antonieta Guzmán, Tari Haahtela, Ramsa Halloum, Eckard Hamelmann, Suleiman Hammadi, Richard Harvey, Joachim Heinrich, Adnan Hejjaoui, Birthe Hellquist-Dahl, Luiana Hernández Velázquez, Mark Hew, Elham Hossny, Peter Howarth, Martin Hrubiško, Yunuen Rocío Huerta Villalobos, Marc Humbert, Michael Hyland, Guido Iaccarino, Moustafa Ibrahim, Maddalena Illario, Natalia Ilyina, Carla Irani, Zhanat Ispayeva, Juan Carlos Ivancevich, Edgardo Jares, Deborah Jarvis, Ewa Jassem, Klemen Jenko, Rubén Darío Jiméneracruz Uscanga, Sebastian Johnston, Guy Joos, Maja Jošt, Kaja Julge, Ki-Suck Jung, Jocelyne Just, Marek Jutel, Igor Kaidashev, Omer Kalayci, Fuat Kalyoncu, Jeni Kapsali, Przemyslaw Kardas, Jussi Karjalainen, Carmela A. Kasala, Michael Katotomichelakis, Bennoor Kazi, Thomas Keil, Paul Keith, Musa Khaitov, Nikolai Khaltaev, You-Young Kim, Jorg Kleine-Tebbe, Ludger Klimek, Bernard Koffi N’Goran, Evangelia Kompoti, Peter Kopač, Gerard Koppelman, Anja Koren Jeverica, Mitja Košnik, Kosta V. Kostov, Marek L. Kowalski, Tanya Kralimarkova, Karmen Kramer Vrščaj, Helga Kraxner, Samo Kreft, Vicky Kritikos, Dmitry Kudlay, Inger Kull, Piotr Kuna, Maciej Kupczyk, Violeta Kvedariene, Marialena Kyriakakou, Nika Lalek, Stephen Lane, Désiree Larenas-Linnemann, Amir Latiff, Susanne Lau, Daniel Laune, Jorge Lavrut, Lan Le, Marcus Lessa, Michael Levin, Jing Li, Philip Lieberman, Giuseppe Liotta, Brian Lipworth, Xuandao Liu, Rommel Lobo, Karin C. Lodrup Carlsen, Carlo Lombardi, Renaud Louis, Stelios Loukidis, Olga Lourenço, Jorge A. Luna Pech, Bojan Madjar, Antoine Magnan, Bassam Mahboub, Alpana Mair, Yassin Mais, Anke-Hilse Maitland van der Zee, Mika Makela, Michael Makris, Hans-Jorgen Malling, Mariana Mandajieva, Patrick Manning, Manolis Manousakis, Pavlos Maragoudakis, Gailen Marshall, Pedro MartinsMartins, Mohammad Reza Masjedi, Jorge F. Máspero, Juan José Matta Campos, Marcus Maurer, Sandra Mavale-Manuel, Cem Meço, Erik Melén, Elisabete Melo-Gomes, Eli O. Meltzer, Enrica Menditto, Andrew Menzies-Gow, Hans Merk, Jean-Pierre Michel, Neven Miculinic, Luís Midão, Florin Mihaltan, Kuitunen Mikael, Nikolaos Mikos, Branislava Milenkovic, Dimitrios Mitsias, Bassem Moalla, Giuliana Moda, María Dolores Mogica Martínez, Yousser Mohammad, Mostafa Moin, Mathieu Molimard, Isabelle Momas, Alessandro Monaco, Steve Montefort, Dory Mora, Mario Morais-Almeida, Ralph Mösges, Badr Eldin Mostafa, Joaquim Mullol, Lars Münter, Antonella Muraro, Ruth Murray, Tihomir Mustakov, Robert Naclerio, Rachel Nadif, Alla Nakonechna, Leyla Namazova-Baranova, Gretchen Navarro-Locsin, Hugo Neffen, Kristof Nekam, Angelos Neou, Laurent Nicod, Verena Niederberger-Leppin, Marek Niedoszytko, Antonio Nieto, Ettore Novellino, Elizabete Nunes, Dieudonné Nyembue, Robyn O’Hehir, Cvetanka Odjakova, Ken Ohta, Yoshitaka Okamoto, Kimi Okubo, Brian Oliver, Gabrielle L. Onorato, Maria Pia Orru, Solange Ouédraogo, Kampadilemba Ouoba, Pier Luigi Paggiaro, Aris Pagkalos, S. P. Palaniappan, Isabella Pali-Schöll, Susanna Palkonen, Stephen Palmer, Carmen Panaitescu Bunu, Petr Panzner, Nikos G. Papadopoulos, Vasilis Papanikolaou, Alberto Papi, Bojidar Paralchev, Giannis Paraskevopoulos, Hae Sim Park, Giovanni Passalacqua, Vincenzo Patella, Ian Pavord, Ruby Pawankar, Soren Pedersen, Susete Peleve, Ana Pereira, Tamara Pérez, Oliver Pfaar, Nhân Pham-Thi, Bernard Pigearias, Isabelle Pin, Konstantina Piskou, Constantinos Pitsios, Kostas Pitsios, Davor Plavec, Dagmar Poethig, Wolfgang Pohl, Antonija Poplas Susic, Todor A. Popov, Fabienne Portejoie, Paul Potter, Lars Poulsen, Alexandra Prados-Torres, Fotis Prarros, David Price, Emmanuel Prokopakis, Robert Puy, Klaus Rabe, Filip Raciborski, Josephine Ramos, Marysia T. Recto, Shereen M. Reda, Frederico Regateiro, Norbert Reider, Sietze Reitsma, Susana Repka-Ramirez, Janet Rimmer, Daniela Rivero Yeverino, José Angelo Rizzo, Carlos Robalo-Cordeiro, Graham Roberts, Nicolas Roche, Mónica Rodríguez González, Eréndira Rodríguez Zagal, Christine Rolland, Regina Roller-Wirnsberger, Miguel Roman Rodriguez, Antonino Romano, Philippe Rombaux, Joel Romualdez, Jose Rosado-Pinto, Nelson Rosario, Lanny Rosenwasser, Menachem Rottem, Philip Rouadi, Nikoleta Rovina, Irma Rozman Sinur, Mauricio Ruiz, Lucy Tania Ruiz Segura, Dermot Ryan, Hironori Sagara, Daiki Sakai, Daiju Sakurai, Wafaa Saleh, Johanna Salimaki, Husain Salina, Konstantinos Samitas, Boleslaw Samolinski, María Guadalupe Sánchez Coronel, Mario Sanchez-Borges, Jaime Sanchez-Lopez, Codrut Sarafoleanu, Faradiba Sarquis Serpa, Joaquin Sastre-Dominguez, Glenis Scadding, Sophie Scheire, Peter Schmid-Grendelmeier, Juan Francisco Schuhl, Holger Schunemann, Maria Schvalbová, Nicola Scichilone, Cecilia Sepúlveda, Elie Serrano, Aziz Sheikh, Mike Shields, Vasil Shishkov, Nikos Siafakas, Alexander Simeonov, Estelle F. Simons, Juan Carlos Sisul, Brigita Sitkauskiene, Ingelbjorg Skrindo, Tanja Soklič Košak, Dirceu Solé, Talant Sooronbaev, Manuel Soto-Martinez, Milan Sova, François Spertini, Otto Spranger, Sofia Stamataki, Lina Stefanaki, Cristiana Stellato, Rafael Stelmach, Peter Sterk, Timo Strandberg, Petra Stute, Abirami Subramaniam, Charlotte Suppli Ulrik, Michael Sutherland, Silvia Sylvestre, Aikaterini Syrigou, Luis Taborda Barata, Nadejda Takovska, Rachel Tan, Frances Tan, Vincent Tan, Ing Ping Tang, Masami Taniguchi, Line Tannert, Jessica Tattersall, Maria do Ceu Teixeira, Carel Thijs, Mike Thomas, Teresa To, Ana Maria Todo-Bom, Alkis Togias, Peter-Valentin Tomazic, Sanna Toppila-Salmi, Elina Toskala, Massimo Triggiani, Nadja Triller, Katja Triller, Ioanna Tsiligianni, Ruxandra Ulmeanu, Jure Urbancic, Marilyn Urrutia Pereira, Martina Vachova, Felipe Valdés, Rudolf Valenta, Marylin Valentin Rostan, Antonio Valero, Arunas Valiulis, Mina Vallianatou, Erkka Valovirta, Michiel Van Eerd, Eric Van Ganse, Marianne van Hage, Olivier Vandenplas, Tuula Vasankari, Dafina Vassileva, Maria Teresa Ventura, Cécilia Vera-Munoz, Dilyana Vicheva, Pakit Vichyanond, Petra Vidgren, Giovanni Viegi, Claus Vogelmeier, Leena Von Hertzen, Theodoros Vontetsianos, Dimitris Vourdas, Martin Wagenmann, Samantha Walker, Dana Wallace, De Yun Wang, Susan Waserman, Magnus Wickman, Sian Williams, Dennis Williams, Nicola Wilson, Kent Woo, John Wright, Piotr Wroczynski, Paraskevi Xepapadaki, Plamen Yakovliev, Masao Yamaguchi, Kwok Yan, Yoke Yeow Yap, Barbara Yawn, Panayiotis Yiallouros, Arzu Yorgancioglu, Shigemi Yoshihara, Ian Young, Osman B. Yusuf, Asghar Zaidi, Fares Zaitoun, Heather Zar, Mario Zernotti, Luo Zhang, Nanshan Zhong, Mihaela Zidarn, Torsten Zuberbier, Celia Zubrinich

**Affiliations:** 1 Charité Universitätsmedizin Berlin, Humboldt-Universität zu Berlin, Berlin, Germany; 2 Department of Dermatology and Allergy, Berlin Institute of Health, Comprehensive Allergy Center, Berlin, Germany; 3 MACVIA-France, Montpellier, France; 4 CHU Montpellier, 273 Avenue d’Occitanie, 34090 Montpellier, France; 5 Centre for Research in Environmental Epidemiology (CREAL), ISGlobAL, Barcelona, Spain; 6 IMIM (Hospital del Mar Research Institute), Barcelona, Spain; 7 Universitat Pompeu Fabra (UPF), Barcelona, Spain; 8 CIBER Epidemiología y Salud Pública (CIBERESP), Barcelona, Spain; 9 Department of Advanced Biomedical Sciences, Federico II University, Naples, Italy; 10 MASK-air, Montpellier, France; 11 Medical Consulting Czarlewski, Levallois, France; 12 Helsinki University, Helsinki, Finland; 13 Swiss Institute of Allergy and Asthma Research (SIAF), University of Zurich, Davos, Switzerland; 14 Department of Geriatrics, Montpellier University Hospital, Montpellier, France; 15 EA 2991, Euromov, University Montpellier, Montpellier, France; 16 Personalized Medicine Clinic Asthma & Allergy, Humanitas University, Humanitas Research Hospital, Rozzano, Milan Italy; 17 Allergy Section, Department of Internal Medicine, Hospital Vall d’Hebron & ARADyAL research network, Barcelona, Spain; 18 ProAR – Nucleo de Excelencia em Asma, Federal University of Bahia, Salvador, Brazil; 19 WHO GARD Planning Group, Salvador, Brazil; 20 Division for Health Innovation, Campania Region, Naples, Italy; 21 Federico II University Hospital Naples (R&D and DISMET), Naples, Italy; 22 Clinica Santa Isabel, Servicio de Alergia e Immunologia, Buenos Aires, Argentina; 23 Department of Clinical Immunology, Wrocław Medical University, Wrocław, Poland; 24 Center for Rhinology and Allergology, Wiesbaden, Germany; 25 Division of Internal Medicine, Asthma and Allergy, Barlicki University Hospital, Medical University of Lodz, Łódź, Poland; 26 KYomed INNOV, Montpellier, France; 27 Center of Excellence in Asthma and Allergy, Médica Sur Clinical Foundation and Hospital, México City, Mexico; 28 Rhinology Unit & Smell Clinic, ENT Department, Hospital Clínic; Clinical & Experimental Respiratory Immunoallergy, IDIBAPS, CIBERES, University of Barcelona, Barcelona, Spain; 29 Division of Infection, Immunity & Respiratory Medicine, Royal Manchester Children’s Hospital, University of Manchester, Manchester, UK; 30 Allergy Department, 2nd Pediatric Clinic, Athens General Children’s Hospital “P&A Kyriakou,”, University of Athens, Athens, Greece; 31 Department of Otorhinolaryngology, Head and Neck Surgery, Section of Rhinology and Allergy, University Hospital Marburg, Phillipps-Universität Marburg, Marburg, Germany; 32 Department of Prevention of Envinronmental Hazards and Allergology, Medical University of Warsaw, Warsaw, Poland; 33 Institute of Clinical Medicine & Institute of Health Sciences, Vilnius University Faculty of Medicine, Vilnius, Lithuania; 34 Department of Pulmonary Diseases, Celal Bayar University, Faculty of Medicine, Manisa, Turkey

**Keywords:** Coronavirus, Diet, Angiotensin-converting enzyme, Antioxidant, Food

## Abstract

Reported COVID-19 deaths in Germany are relatively low as compared to many European countries. Among the several explanations proposed, an early and large testing of the population was put forward. Most current debates on COVID-19 focus on the differences among countries, but little attention has been given to regional differences and diet. The low-death rate European countries (e.g. Austria, Baltic States, Czech Republic, Finland, Norway, Poland, Slovakia) have used different quarantine and/or confinement times and methods and none have performed as many early tests as Germany. Among other factors that may be significant are the dietary habits. It seems that some foods largely used in these countries may reduce angiotensin-converting enzyme activity or are anti-oxidants. Among the many possible areas of research, it might be important to understand diet and angiotensin-converting enzyme-2 (ACE2) levels in populations with different COVID-19 death rates since dietary interventions may be of great benefit.

## Introduction

A novel strain of human coronaviruses, the severe acute respiratory syndrome coronavirus 2 (SARS-CoV-2), named by the International Committee on Taxonomy of Viruses (ICTV) [1], has emerged and caused an infectious disease referred to as “coronavirus disease 2019” (COVID-19) by the World Health Organization (WHO) [2]. COVID-19 has aggressively spread across the globe and over 160,000 deaths have been reported. However, there appears to be high- and low-death rate countries.

After the outbreak in China, COVID-19 has also affected Europe after becoming a pandemic. Interestingly, there is large variability across European countries in both incidence and mortality, and most current debates on COVID-19 focus on the differences among countries. German fatalities are strikingly low as compared to many European countries. Among the several explanations proposed, an early and large testing of the population was put forward [3].

However, little attention has been given to regional differences and diet [4].

## Biases to be considered

According to the Johns Hopkins coronavirus resource center (https://coronavirus.jhu.edu), one of the most important ways of measuring the burden of COVID-19 is mortality. However, death rates are assessed differently between countries and there are many biases that are almost impossible to assess. Differences in the mortality rates depend on the characteristics of the health care system, the reporting method, whether or not deaths outside the hospital have been counted and other factors, many of which remain unknown. Countries throughout the world have reported very different case fatality ratios—the number of deaths divided by the number of confirmed cases—but these numbers cannot be compared at all due to biases. On the other hand, for many countries, the methodology reporting death rates in the different regions is standardized across the country.

## European data on death rates per million inhabitants

**Fig. 1:**
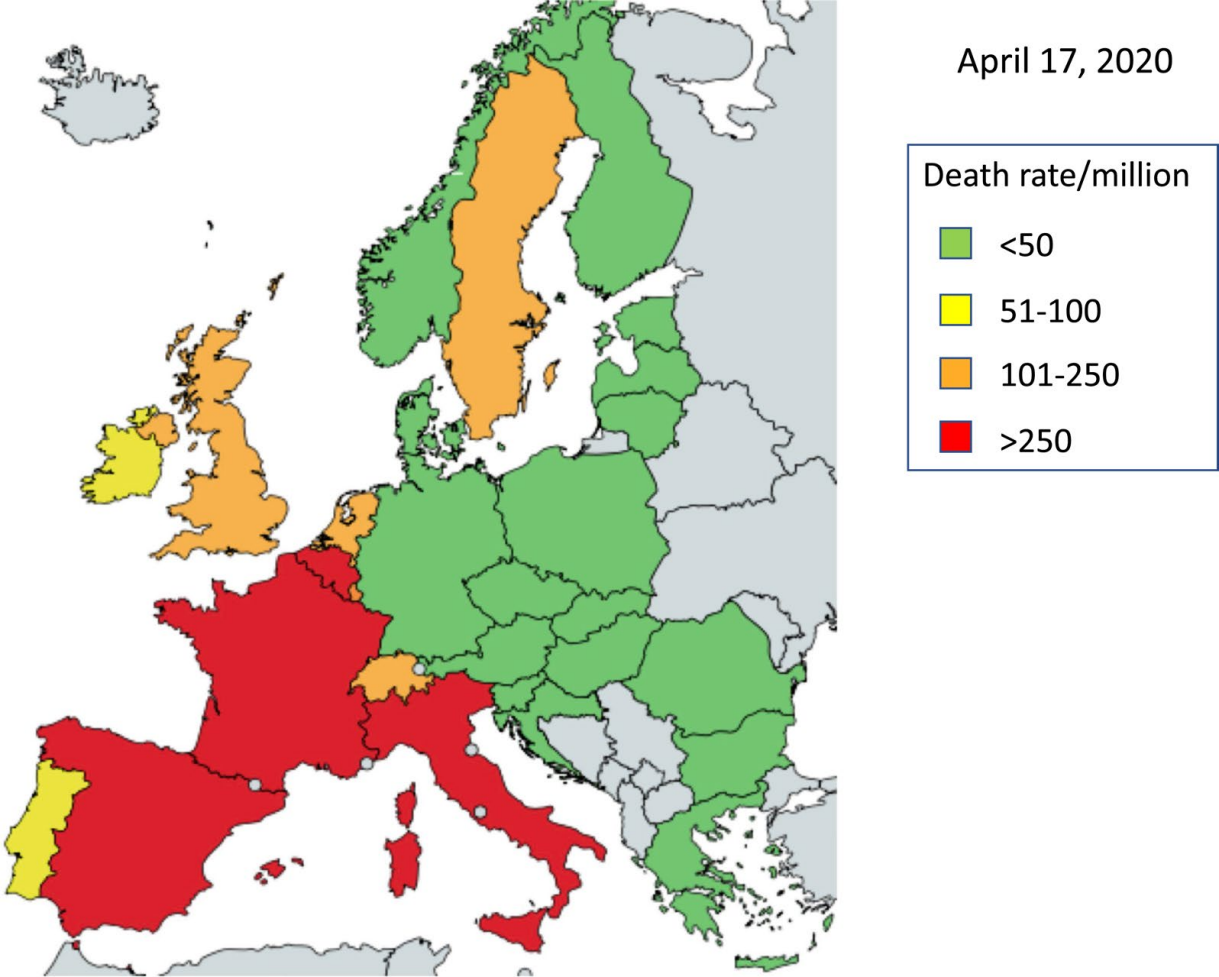
COVID-19 deaths per million inhabitants in Europe (April 17, 2020). For France, deaths included hospital and extra-hospital deaths

We used the Johns Hopkins coronavirus resource center to assess death rates at the national level (https://coronavirus.jhu.edu). The current death rate per million people in Europe shows different trends. Germany has a low death rate, but Austria, the Czech Republic, Poland, Slovakia, the Baltic States and Finland have similar or lower rates. On the other hand, Belgium, France, Italy, Spain and the UK have higher rates (Fig. 1).

**Fig. 2:**
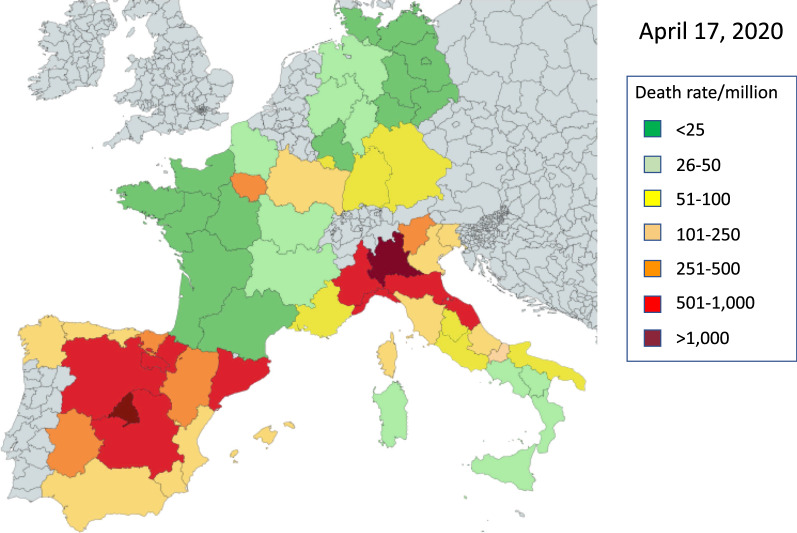
Regional COVID-19 death rates per million in four European countries

Large differences exist when assessing death rates within a country. In Germany, Bavaria started the earliest tests but was and still is the most affected region (Fig. 2). Death rates per million range from 8 in Mecklenburg-Vorpommern to 87 in Bavaria.

**Fig. 3:**
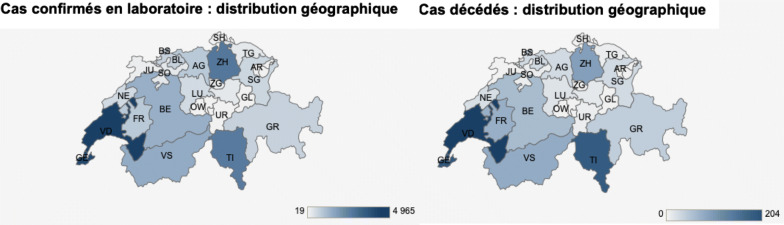
COVID-19 rates in Switzerland (Office fédéral de la santé publique). Cas confirmés en laboratoire (laboratory confirmed cases), distribution géographique (geographical distibution), cas décédés (death rate)

In Switzerland, the French and Italian speaking cantons have a far higher death rate than the German-speaking ones (Fig. 3) (Office fédéral de la santé publique, Switzerland, https://www.bag.admin.ch/bag/fr/home.html).

In high-rate countries such as Spain, large variations also exist within the country, but the numbers range from 115 in Murcia to over 1000 in Madrid.

## Is diet partly involved in different death rates between countries?

Most diseases exhibit large geographical variations which frequently remain unexplained despite abundant research [5]. COVID-19 will not be an exception. Though the more relevant factors are likely to be seasonal variations, immunity, cross-immunity, intensity, timing of measures [6], type, onset, duration and measures of protection, other factors like environment or nutrition should not be overlooked. Obesity, a risk factor of mortality in COVID-19, suggests the importance of nutrition [7].

The “low-rate” European countries have used different quarantine and/or confinement times and methods and none have performed as many early tests as Germany. Thus, although the German testing approach is very important [3], other factors may also be significant.

### Immunity in COVID-19 and ageing

Although there are large differences between countries in death rates, the age-dependent severity of COVID-19 is similar between Asian, European and American countries. The rate of deaths is increased in the older population. Globally, there are risk factors for death including obesity and type 2 diabetes.

A strong relationship between hyperglycemia, impaired insulin pathway, and cardiovascular disease in type 2 diabetes is linked to oxidative stress and inflammation [8]. Lipid metabolism has an important role to play in obesity, diabetes and its multi-morbidities, and the ageing process [9]. Dietary fatty acids have a significant role in immune responses [10].

Many foods have an antioxidant activity [11–13]. Resveratrol, present in many foods [14], is an inhibitor of MERS-Coronavirus infection [15].

### Angiotensin-converting enzyme 2 (ACE-2)

The angiotensin-converting enzyme (ACE2) has multiple physiological roles: a negative regulator of the renin-angiotensin system, facilitator of amino acid transport, and the SARS-CoV and SARS-CoV-2 receptor [16]. ACE converts angiotensin I to angiotensin II but ACE2 catalyses the conversion of angiotensin II to angiotensin and is also the main entry point for coronavirus 2 into cells.

Differences between countries in ACE have been associated with genetic patterns. The *ACE* D allele increased risk of vasculitis [17] or hypertension [18]. The *ACE* I/D polymorphism is involved in the onset of type 2 diabetes [19] and might be associated with susceptibility to peripheral vascular diseases in the Asian population [20].

However, dietary patterns have a strong effect on ACE levels. A high-saturated fat diet increases ACE [21]. Many foods have an ACE-inhibitory activity [22–24]. Anti-oxidant activities and ACE inhibition have been largely found in many foods [25]. Moreover, ACE levels in blood are highly and rapidly sensitive to food intake [26].

Identifying whether countries with high or low ACE activity have different death rates would be of great interest in understanding the clinical importance of interventions. However, the available evidence, in particular from human studies, does not seem to support the hypothesis that inhibitors of ACE or renin-angiotensin–aldosterone (ACEI/ARB) drugs increase the ACE2 expression and the risk of COVID-19 [27]. This might suggest that changes in ACE expression (inhibition/stimulation) might not be as relevant as previously thought and other diet-related changes might be more (or equally) important.

### Possible interactions between diet and COVID-19 death rate

Germany, Austria, Croatia, the Czech Republic, Poland, Slovakia, the Baltic States and German-speaking Swiss cantons exhibit lower COVID-19 mortality rates than France, Italy, Spain, and the French and Italian speaking Swiss cantons. Among many factors, diet differs considerably between these low- or high-mortality countries.

It appears that death rates in Germany are higher in the two Southern Regions as well as in Saarland than elsewhere. Baden-Wurttemberg and Saarland are in close contact with Alsace (France), and the higher infection rate may be due to the high cross-border traffic of the French. However, this was not the case for Rhineland-Palatinate (lower death rate), possibly because the East Region of France was contaminated later. In addition, Saarland is a special case as half of the deaths, unlike in the other German states, occurred in only a few long-term care facilities where a high number of people were infected in a short time and all deaths during the episode were attributed to Corona without autopsies being made. This potential French-based contamination does not apply for Bavaria (earliest German region to be contaminated and highest death rate). Diet differs within Germany, the southern states traditionally having a higher fat-rich diet. Diet is not normally distributed within country/region, which can be an additional argument in favour of the uneven distribution of mortality.

Nutrition may therefore play a role in the immune defense against COVID-19 and may explain some of the differences seen in COVID-19 across Europe. It will be needed to test dietary differences between low and high-rate countries. Foods with potent antioxidant or anti ACE activity—like uncooked or fermented cabbage [28–30]—are largely consumed in low-death rate European countries, Korea and Taiwan, and might be considered in the low prevalence of deaths.

Although it is difficult to compare health systems and death reporting across European countries, Bulgaria, Greece and Romania have very low death rates. This might also be associated with diet since cabbage (Romania) and fermented milk (Bulgaria and Greece) are common foods. The latter food is a known ACE natural inhibitor [31]. Turkey, another apparently low-death rate country, also consumes a lot of cabbage and fermented milk products.

Another example may be the food supply chain. The increasing availability of foods from big retail is a revolutionary event that has impacted crops (favouring those that have the best ratio of effectiveness over costs of production) and health at a population-size level. In particular, such a change in food availability has altered alimentary habits—promoting sugar-enriched, vitamin-depauperated foods—and has become one of the causes of the obesity epidemic, especially among adolescents. These foods come from centralized farms in selected areas of the world that are distributed around the planet, elongating the supply chain of food. The impact of long supply chain of food on health is measurable by an increase in metabolic syndrome and insulin resistance [32]. Therefore, rural areas that are more prone to short supply food may have been able to better tolerate the COVID-19 pandemia, with a lower death toll. These considerations may be partly involved in lower death rates in Southern Italy compared to the Northern part.

## Conclusions

Understanding the within and between country differences in COVID-19 will be of paramount importance in understanding COVID-19 risk and protective factors, and will eventually help to control the epidemics.

We acknowledge that many factors may play a role in the extension and severity of COVID-19, such as trained immunity of the population, early and fast education, rapid organization and adaptation of the hospitals and the public, preparedness for pandemics and public hygiene. Diet represents only one of the possible causes of the COVID-19 epidemic and its importance needs to be better assessed.

## Data Availability

Not applicable.

## Abbreviations

ACE: Angiotensin converting enzyme
COVID-19: Coronavirus 19 disease
MERS: Middle East Respiratory Syndrome
SARS: Severe acute respiratory syndrome
SARS-Cov-2: Severe acute respiratory syndrome coronavirus 2
