# Correction to: Is diet partly responsible for differences in COVID-19 death rates between and within countries?

**DOI:** 10.1186/s13601-020-00351-w

**Published:** 2020-10-26

**Authors:** Jean Bousquet, Josep M. Anto, Guido Iaccarino, Wienczyslawa Czarlewski, Tari Haahtela, Aram Anto, Cezmi A. Akdis, Hubert Blain, G. Walter Canonica, Victoria Cardona, Alvaro A. Cruz, Maddalena Illario, Juan Carlos Ivancevich, Marek Jutel, Ludger Klimek, Piotr Kuna, Daniel Laune, Désirée Larenas‑Linnemann, Joaquim Mullol, Nikos G. Papadopoulos, Oliver Pfaar, Boleslaw Samolinski, Arunas Valiulis, Arzu Yorgancioglu, Torsten Zuberbier, The ARIA group, Amir Hamzah Abdul Latiff, Baharudin Abdullah, Werner Aberer, Nancy Abusada, Ian Adcock, Alejandro Afani, Ioana Agache, Xenofon Aggelidis, Jenifer Agustin, Cezmi Akdis, Mübeccel Akdis, Mona Al-Ahmad, Abou Al-Zahab Bassam, Oscar Aldrey-Palacios, Emilio Alvarez Cuesta, Ashraf Alzaabi, Salma Amad, Gene Ambrocio, Isabella Annesi-Maesano, Ignacio Ansotegui, Josep Anto, Hasan Arshad, Maria Cristina Artesani, Estrella Asayag, Francesca Avolio, Khuzama Azhari, Ilaria Baiardini, Nissera Bajrović, Petros Bakakos, Sergio Bakeyala Mongono, Christine Balotro-Torres, Sergio Barba, Cristina Barbara, Elsa Barbosa, Bruno Barreto, Joan Bartra, Eric D. Bateman, Lkhagvaa Battur, Anna Bedbrook, Martín Bedolla Barajas, Bianca Beghé, Elizabeth Bel, Ali Ben Kheder, Mikael Benson, Camelia Berghea, Karl-Christian Bergmann, David Bernstein, Mike Bewick, Slawomir Bialek, Artur Białoszewski, Thomas Bieber, Nils Billo, Maria Beatrice Bilo, Carsten Bindslev-Jensen, Leif Bjermer, Hubert Blain, Malgorzata Bochenska Marciniak, Christine Bond, Attilio Boner, Matteo Bonini, Sergio Bonini, Sinthia Bosnic-Anticevich, Isabelle Bosse, Sofia Botskariova, Jacques Bouchard, Louis-Philippe Boulet, Rodolphe Bourret, Philippe Bousquet, Fulvio Braido, Andrew Briggs, Christopher Brightling, Jan Brozek, Roland Buhl, Roxana Bumbacea, María Teresa Burguete Cabañas, Andrew Bush, William W. Busse, Jeroen Buters, Fernan Caballero-Fonseca, Moïses A. Calderon, Mario Calvo, Paulo Camargos, Thierry Camuzat, Antonio Cano, G. Walter Canonica, Arnaldo Capriles-Hulett, Luis Caraballo, Vicky Cardona, Kai-Hakon Carlsen, Jorge Caro, Warner Carr, Fredelita Carreon-Asuncion, Ana Maria Carriazo, Thomas Casale, Mary Ann Castor, Elizabeth Castro, Lorenzo Cecchi, Alfonso Cepeda Sarabia, Ramanathan Chandrasekharan, Yoon-Suk Chang, Victoria Chato-Andeza, Lida Chatzi, Christina Chatzidaki, Niels H. Chavannes, Yuzhi Chen, Lei Cheng, Tomas Chivato, Ekaterine Chkhartishvili, George Christoff, Henry Chrystyn, Derek K. Chu, Antonio Chua, Alexander Chuchalin, Kian Fan Chung, Alberto Cicerán, Cemal Cingi, Giorgio Ciprandi, Ieva Cirule, Ana Carla Coelho, Jannis Constantinidis, Jaime Correia de Sousa, Elisio Costa, David Costa, María del Carmen Costa Domínguez, André Coste, Linda Cox, Alvaro A. Cruz, John Cullen, Adnan Custovic, Biljana Cvetkovski, Wienczyslawa Czarlewski, Gennaro D’Amato, Jane da Silva, Ronald Dahl, Sven-Erik Dahlen, Vasilis Daniilidis, Louei Darjazini Nahhas, Ulf Darsow, Frédéric de Blay, Eloisa De Guia, Chato de los Santos, Esteban De Manuel Keenoy, Govert De Vries, Diana Deleanu, Pascal Demoly, Judah Denburg, Philippe Devillier, Alain Didier, Maria Dimou, Anh Tuan Dinh-Xuan, Ratko Djukanovic, Dejan Dokic, Margarita Gabriela Domínguez Silva, Habib Douagui, Nikolaos Douladiris, Maria Doulaptsi, Gérard Dray, Ruta Dubakiene, Stephen Durham, Mark Dykewicz, Didier Ebo, Natalija Edelbaher, Patrik Eklund, Yehia El-Gamal, Zeinab A. El-Sayed, Shereen S. El-Sayed, Magda El-Seify, Regina Emuzyte, Lourdes Enecilla, Heidilita Espinoza, Jesús Guillermo Espinoza Contreras, John Farrell, Lenora Fernandez, Antje Fink Wagner, Alessandro Fiocchi, Wytske J. Fokkens, Jean-François Fontaine, Francesco Forastiere, Jose Miguel Fuentes Pèrez, Emily Gaerlan–Resureccion, Mina Gaga, José Luis Gálvez Romero, Amiran Gamkrelidze, Alexis Garcia, Cecilia Yvonne García Cobas, María de la Luz Hortensia García Cruz, Jacques Gayraud, Bilun Gemicioglu, Sonya Genova, José Gereda, Roy Gerth van Wijk, Maximiliano Gomez, Sandra González Diaz, Maia Gotua, Christos Grigoreas, Ineta Grisle, Marta Guidacci, Nick Guldemond, Zdenek Gutter, Antonieta Guzmán, Tari Haahtela, Ramsa Halloum, Eckard Hamelmann, Suleiman Hammadi, Richard Harvey, Joachim Heinrich, Adnan Hejjaoui, Birthe Hellquist-Dahl, Luiana Hernández Velázquez, Mark Hew, Elham Hossny, Peter Howarth, Martin Hrubiško, Yunuen Rocío Huerta Villalobos, Marc Humbert, Michael Hyland, Guido Iaccarino, Moustafa Ibrahim, Maddalena Illario, Natalia Ilyina, Carla Irani, Zhanat Ispayeva, Juan Carlos Ivancevich, Edgardo Jares, Deborah Jarvis, Ewa Jassem, Klemen Jenko, Rubén Darío Jiméneracruz Uscanga, Sebastian Johnston, Guy Joos, Maja Jošt, Kaja Julge, Ki-Suck Jung, Jocelyne Just, Marek Jutel, Igor Kaidashev, Omer Kalayci, Fuat Kalyoncu, Jeni Kapsali, Przemyslaw Kardas, Jussi Karjalainen, Carmela A. Kasala, Michael Katotomichelakis, Bennoor Kazi, Thomas Keil, Paul Keith, Musa Khaitov, Nikolai Khaltaev, You-Young Kim, Jorg Kleine-Tebbe, Ludger Klimek, Bernard Koffi N’Goran, Evangelia Kompoti, Peter Kopač, Gerard Koppelman, Anja Koren Jeverica, Mitja Košnik, Kosta V. Kostov, Marek L. Kowalski, Tanya Kralimarkova, Karmen Kramer Vrščaj, Helga Kraxner, Samo Kreft, Vicky Kritikos, Dmitry Kudlay, Inger Kull, Piotr Kuna, Maciej Kupczyk, Violeta Kvedariene, Marialena Kyriakakou, Nika Lalek, Stephen Lane, Désiree Larenas-Linnemann, Amir Latiff, Susanne Lau, Daniel Laune, Jorge Lavrut, Lan Le, Marcus Lessa, Michael Levin, Jing Li, Philip Lieberman, Giuseppe Liotta, Brian Lipworth, Xuandao Liu, Rommel Lobo, Karin C. Lodrup Carlsen, Carlo Lombardi, Renaud Louis, Stelios Loukidis, Olga Lourenço, Jorge A. Luna Pech, Bojan Madjar, Antoine Magnan, Bassam Mahboub, Alpana Mair, Yassin Mais, Anke-Hilse Maitland van der Zee, Mika Makela, Michael Makris, Hans-Jorgen Malling, Mariana Mandajieva, Patrick Manning, Manolis Manousakis, Pavlos Maragoudakis, Gailen Marshall, Pedro MartinsMartins, Mohammad Reza Masjedi, Jorge F. Máspero, Juan José Matta Campos, Marcus Maurer, Sandra Mavale-Manuel, Cem Meço, Erik Melén, Elisabete Melo-Gomes, Eli O. Meltzer, Enrica Menditto, Andrew Menzies-Gow, Hans Merk, Jean-Pierre Michel, Neven Miculinic, Luís Midão, Florin Mihaltan, Kuitunen Mikael, Nikolaos Mikos, Branislava Milenkovic, Dimitrios Mitsias, Bassem Moalla, Giuliana Moda, María Dolores Mogica Martínez, Yousser Mohammad, Mostafa Moin, Mathieu Molimard, Isabelle Momas, Alessandro Monaco, Steve Montefort, Dory Mora, Mario Morais-Almeida, Ralph Mösges, Badr Eldin Mostafa, Joaquim Mullol, Lars Münter, Antonella Muraro, Ruth Murray, Tihomir Mustakov, Robert Naclerio, Rachel Nadif, Alla Nakonechna, Leyla Namazova-Baranova, Gretchen Navarro-Locsin, Hugo Neffen, Kristof Nekam, Angelos Neou, Laurent Nicod, Verena Niederberger-Leppin, Marek Niedoszytko, Antonio Nieto, Ettore Novellino, Elizabete Nunes, Dieudonné Nyembue, Robyn O’Hehir, Cvetanka Odjakova, Ken Ohta, Yoshitaka Okamoto, Kimi Okubo, Brian Oliver, Gabrielle L. Onorato, Maria Pia Orru, Solange Ouédraogo, Kampadilemba Ouoba, Pier Luigi Paggiaro, Aris Pagkalos, S. P. Palaniappan, Isabella Pali-Schöll, Susanna Palkonen, Stephen Palmer, Carmen Panaitescu Bunu, Petr Panzner, Nikos G. Papadopoulos, Vasilis Papanikolaou, Alberto Papi, Bojidar Paralchev, Giannis Paraskevopoulos, Hae Sim Park, Giovanni Passalacqua, Vincenzo Patella, Ian Pavord, Ruby Pawankar, Soren Pedersen, Susete Peleve, Ana Pereira, Tamara Pérez, Oliver Pfaar, Nhân Pham-Thi, Bernard Pigearias, Isabelle Pin, Konstantina Piskou, Constantinos Pitsios, Kostas Pitsios, Davor Plavec, Dagmar Poethig, Wolfgang Pohl, Antonija Poplas Susic, Todor A. Popov, Fabienne Portejoie, Paul Potter, Lars Poulsen, Alexandra Prados-Torres, Fotis Prarros, David Price, Emmanuel Prokopakis, Robert Puy, Klaus Rabe, Filip Raciborski, Josephine Ramos, Marysia T. Recto, Shereen M. Reda, Frederico Regateiro, Norbert Reider, Sietze Reitsma, Susana Repka-Ramirez, Janet Rimmer, Daniela Rivero Yeverino, José Angelo Rizzo, Carlos Robalo-Cordeiro, Graham Roberts, Nicolas Roche, Mónica Rodríguez González, Eréndira Rodríguez Zagal, Christine Rolland, Regina Roller-Wirnsberger, Miguel Roman Rodriguez, Antonino Romano, Philippe Rombaux, Joel Romualdez, Jose Rosado-Pinto, Nelson Rosario, Lanny Rosenwasser, Menachem Rottem, Philip Rouadi, Nikoleta Rovina, Irma Rozman Sinur, Mauricio Ruiz, Lucy Tania Ruiz Segura, Dermot Ryan, Hironori Sagara, Daiki Sakai, Daiju Sakurai, Wafaa Saleh, Johanna Salimaki, Husain Salina, Konstantinos Samitas, Boleslaw Samolinski, María Guadalupe Sánchez Coronel, Mario Sanchez-Borges, Jaime Sanchez-Lopez, Codrut Sarafoleanu, Faradiba Sarquis Serpa, Joaquin Sastre-Dominguez, Glenis Scadding, Sophie Scheire, Peter Schmid-Grendelmeier, Juan Francisco Schuhl, Holger Schunemann, Maria Schvalbová, Nicola Scichilone, Cecilia Sepúlveda, Elie Serrano, Aziz Sheikh, Mike Shields, Vasil Shishkov, Nikos Siafakas, Alexander Simeonov, Estelle F. Simons, Juan Carlos Sisul, Brigita Sitkauskiene, Ingelbjorg Skrindo, Tanja Soklič Košak, Dirceu Solé, Talant Sooronbaev, Manuel Soto-Martinez, Milan Sova, François Spertini, Otto Spranger, Sofia Stamataki, Lina Stefanaki, Cristiana Stellato, Rafael Stelmach, Peter Sterk, Timo Strandberg, Petra Stute, Abirami Subramaniam, Charlotte Suppli Ulrik, Michael Sutherland, Silvia Sylvestre, Aikaterini Syrigou, Luis Taborda Barata, Nadejda Takovska, Rachel Tan, Frances Tan, Vincent Tan, Ing Ping Tang, Masami Taniguchi, Line Tannert, Jessica Tattersall, Maria do Ceu Teixeira, Carel Thijs, Mike Thomas, Teresa To, Ana Maria Todo-Bom, Alkis Togias, Peter-Valentin Tomazic, Sanna Toppila-Salmi, Elina Toskala, Massimo Triggiani, Nadja Triller, Katja Triller, Ioanna Tsiligianni, Ruxandra Ulmeanu, Jure Urbancic, Marilyn Urrutia Pereira, Martina Vachova, Felipe Valdés, Rudolf Valenta, Marylin Valentin Rostan, Antonio Valero, Arunas Valiulis, Mina Vallianatou, Erkka Valovirta, Michiel Van Eerd, Eric Van Ganse, Marianne van Hage, Olivier Vandenplas, Tuula Vasankari, Dafina Vassileva, Maria Teresa Ventura, Cécilia Vera-Munoz, Dilyana Vicheva, Pakit Vichyanond, Petra Vidgren, Giovanni Viegi, Claus Vogelmeier, Leena Von Hertzen, Theodoros Vontetsianos, Dimitris Vourdas, Martin Wagenmann, Samantha Walker, Dana Wallace, De Yun Wang, Susan Waserman, Magnus Wickman, Sian Williams, Dennis Williams, Nicola Wilson, Kent Woo, John Wright, Piotr Wroczynski, Paraskevi Xepapadaki, Plamen Yakovliev, Masao Yamaguchi, Kwok Yan, Yoke Yeow Yap, Barbara Yawn, Panayiotis Yiallouros, Arzu Yorgancioglu, Shigemi Yoshihara, Ian Young, Osman B. Yusuf, Asghar Zaidi, Fares Zaitoun, Heather Zar, Mario Zernotti, Luo Zhang, Nanshan Zhong, Mihaela Zidarn, Torsten Zuberbier, Celia Zubrinich

**Affiliations:** 1 Charité Universitätsmedizin Berlin, Humboldt-Universität zu Berlin, Berlin, Germany; 2 Department of Dermatology and Allergy, Berlin Institute of Health, Comprehensive Allergy Center, Berlin, Germany; 3 MACVIA-France, Montpellier, France; 4 CHU Montpellier, 273 Avenue d’Occitanie, 34090 Montpellier, France; 5 Centre for Research in Environmental Epidemiology (CREAL), ISGlobAL, Barcelona, Spain; 6 IMIM (Hospital del Mar Research Institute), Barcelona, Spain; 7 Universitat Pompeu Fabra (UPF), Barcelona, Spain; 8 CIBER Epidemiología y Salud Pública (CIBERESP), Barcelona, Spain; 9 Department of Advanced Biomedical Sciences, Federico II University, Naples, Italy; 10 MASK-air, Montpellier, France; 11 Medical Consulting Czarlewski, Levallois, France; 12 Helsinki University, Helsinki, Finland; 13 Swiss Institute of Allergy and Asthma Research (SIAF), University of Zurich, Davos, Switzerland; 14 Department of Geriatrics, Montpellier University Hospital, Montpellier, France; 15 EA 2991, Euromov, University Montpellier, Montpellier, France; 16 Personalized Medicine, Asthma and Allergy – Humanitas Clinical and Research Center – IRCCS, Rozzano, MI Italy; 17 Department of Biomedical Sciences, Humanitas University, Pieve Emanuele, MI Italy; 18 Allergy Section, Department of Internal Medicin, Hospital Vall d’Hebron &amp; ARADyAL Research Network, Barcelona, Spain; 19 ProAR – Nucleo de Excelencia em Asma, Federal University of Bahia, Salvador, Brazil; 20 WHO GARD Planning Group, Salvador, Brazil; 21 Division for Health Innovation, Campania Region, Naples, Italy; 22 Federico II University Hospital Naples (R&D and DISMET), Naples, Italy; 23 Clinica Santa Isabel, Servicio de Alergia e Immunologia, Buenos Aires, Argentina; 24 Department of Clinical Immunology, Wrocław Medical University, Wrocław, Poland; 25 Center for Rhinology and Allergology, Wiesbaden, Germany; 26 Division of Internal Medicine, Asthma and Allergy, Barlicki University Hospital, Medical University of Lodz, Łódź, Poland; 27 KYomed INNOV, Montpellier, France; 28 Center of Excellence in Asthma and Allergy, Médica Sur Clinical Foundation and Hospital, México City, Mexico; 29 Rhinology Unit & Smell Clinic, ENT Department, Hospital Clínic; Clinical & Experimental Respiratory Immunoallergy, IDIBAPS, CIBERES, University of Barcelona, Barcelona, Spain; 30 Division of Infection, Immunity & Respiratory Medicine, Royal Manchester Children’s Hospital, University of Manchester, Manchester, UK; 31 Allergy Department, 2nd Pediatric Clinic, Athens General Children’s Hospital “P&amp;A Kyriakou,”, University of Athens, Athens, Greece; 32 Department of Otorhinolaryngology, Head and Neck Surgery, Section of Rhinology and Allergy, University Hospital Marburg, Phillipps-Universität Marburg, Marburg, Germany; 33 Department of Prevention of Envinronmental Hazards and Allergology, Medical University of Warsaw, Warsaw, Poland; 34 Institute of Clinical Medicine & Institute of Health Sciences, Vilnius University Faculty of Medicine, Vilnius, Lithuania; 35 Department of Pulmonary Diseases, Celal Bayar University, Faculty of Medicine, Manisa, Turkey

## Correction to: Clin Transl Allergy (2020) 10:16. 10.1186/s13601-020-00323-0

Following publication of the original article [1], the authors identified an error in the affiliation list. The affiliation of author G. Walter Canonica should have been split up into two affiliations:

- Personalized Medicine, Asthma and Allergy – Humanitas Clinical and Research Center – IRCCS, Rozzano (MI), Italy
- Department of Biomedical Sciences, Humanitas University, Pieve Emanuele (MI), Italy

The corrected affiliation list is reflected in this Correction.
